# Sensory adaptation at ribbon synapses in the zebrafish lateral line

**DOI:** 10.1113/JP281646

**Published:** 2021-07-09

**Authors:** Francesca De Faveri, Walter Marcotti, Federico Ceriani

**Affiliations:** 1 Department of Biomedical Science, University of Sheffield, Sheffield, S10 2TN, UK; 2 Neuroscience Institute, University of Sheffield, Sheffield, S10 2TN, UK

## Abstract

Adaptation is used by sensory systems to adjust continuously their sensitivity to match changes in environmental stimuli. In the auditory and vestibular systems, the release properties of glutamate-containing vesicles at the hair-cell ribbon synapses play a crucial role in sensory adaptation, thus shaping the neural response to sustained stimulation. How ribbon synapses regulate the release of glutamate and how they modulate afferent responses *in vivo* is still largely unknown. Here, we have used two-photon imaging and electrophysiology to investigate the synaptic transfer characteristics of the hair cells in the context of sensory adaptation in live zebrafish. Prolonged and repeated water-jet stimulation of the hair-cell stereociliary bundles caused adaptation of the action potential firing rate elicited in the afferent neurons. By monitoring glutamate at ribbon synapses using time-lapse imaging, we identified two kinetically distinct release components: a rapid response that was exhausted within 50-100 ms and a slower and sustained response lasting the entire stimulation. After repeated stimulations, the recovery of the fast component followed a biphasic time course. Depression of glutamate release was largely responsible for the rapid firing rate adaptation recorded in the afferent neurons. However, postsynaptic Ca^2+^ responses had a slower recovery time course than that of glutamate release, indicating that they are also likely to contribute to the afferent firing adaptation. Hair cells also exhibited a form of adaptation during inhibitory bundle stimulations. We conclude that hair cells have optimised their synaptic machinery to encode prolonged stimuli and to maintain their sensitivity to new incoming stimuli.

## Abbreviations

PLLg: posterior lateral line ganglion
RRP: readily releasable pool of vesicles
SRP: secondarily releasable pool of vesicles
IHCs: inner hair cells
MET current: mechanoelectrical transducer current
*P*_o_: open probability
dpf: days post-fertilisation
fps: frames per second

## Introduction

In auditory, vestibular and lateral line systems, the sensory receptors hair cells play a crucial role in ensuring that the relevant features of acoustic and hydrodynamic stimuli, such as intensity and timing, are efficiently encoded in order to provide the brain with a precise representation of the environment. Similar to other sensory modalities, adaptation of hair-cell response to sustained stimuli is critical to preserve the encoding capabilities of the system over a wide dynamic range, preventing saturation of postsynaptic processing and ensuring that their dynamic range rescales to match the input domain (e.g. Torre *et al*. 1995; Pérez-Gonzalez & Malmierca, 2014). In the mammalian auditory system, the adaptation of the firing rate of auditory neurons is primarily linked to the biophysical properties of the hair-cell ribbon synapses (Goutman & Glowatski, 2007, Goutman, 2017, Moser & Beutner, 2000). The ribbon is a specialised presynaptic structure capable of tethering numerous glutamate-filled synaptic vesicles at the release sites (Sterling & Matthews, 2005), which allow hair cells to drive high rates of sustained synaptic transmission (mammals: Glowatzki & Fuchs, 2002; Goutman & Glowatzki, 2007; Neef *et al*. 2007; zebrafish: Lv *et al*. 2016; Pichler & Lagnado, 2019) and to maintain the temporal precision of sensory information (Fuchs, 2005). Among ribbon-associated vesicles, those closer to the plasma membrane are thought to form the readily releasable pool (RRP), which mediates the fastest component of exocytosis (von Gersdorff & Matthews, 1997; Moser & Beutner, 2000; Johnson *et al*. 2008; Johnson *et al*. 2017). The remaining vesicles, located further away from Ca^2+^ channels, are thought to form the secondarily releasable pool (SRP), underpinning the slower, sustained phase of release. Depletion of the RRP, together with presynaptic Ca^2+^ channel inactivation and postsynaptic AMPA receptor desensitisation, have been proposed as mechanisms contributing to the firing rate adaptation in auditory neurons (Goutman & Glowatski, 2007, Goutman, 2017, Moser & Beutner, 2000, Spassova *et al*. 2004).

In recent years, the zebrafish (*Danio rerio*) is becoming an important model to study hair-cell development and function in an intact organism *in vivo* (Obholzer *et al*. 2008; Trapani *et al*. 2011, Ricci *et al*. 2013; Haehnel-Taguchi *et al*. 2014, Olt *et al*. 2014; Levi *et al*. 2015, Olt *et al*. 2016, Pichler & Lagnado, 2019; Wong *et al*. 2019). The zebrafish has hair cells not only in the inner ear, but also in the end organs (neuromasts) of the lateral line system (Nicolson, 2005). Neuromasts of the posterior lateral line, located along the body and tail of the fish, show planar polarity along the anterior-posterior or dorsal-ventral axis of the zebrafish, with two populations of hair cells responding to stimuli of opposite direction (Nicolson *et al*. 1998; Kindt *et al*. 2012). The mechanosensitive stereociliary bundles of the hair cells, which protrude from their apical pole, are embedded in a gelatinous cupula that is in direct contact with the external fluid. Deflection of the hair bundles towards the kinocilium opens the mechanoelectrical transducer (MET) channels and depolarises the hair cells, leading to the opening of CaV1.3 Ca^2+^ channels (Sheets *et al*. 2012). The increased intracellular Ca^2+^ concentration triggers the release of glutamate-filled vesicles and the activation of glutamate receptors at the afferent terminals (Sebe *et al*. 2017). These afferent fibres contact hair cells of the same direction of sensitivity from up to three neuromasts (Nagiel *et al*. 2008, Faucherre *et al*. 2009, Haehnel-Taguchi *et al*. 2014), thus integrating information from multiple hair cells. Compared to mammalian inner hair cells (IHCs), zebrafish lateral line hair cells possess less synaptic ribbons (zebrafish, 2 to 5 per cell: Obholzer *et al*. 2008, Lv *et al*. 2016; mouse, 5 to 20 per cell: Meyer *et al*. 2009) and a lower total number of vesicles available for release during stimulation (~80 vesicles/1 s stimuli in the lateral line hair cells: Olt *et al*. 2014; ~4000 vesicles/1 s stimuli: Johnson *et al*. 2005; Meyer *et al*. 2009). Despite the different number of ribbons and vesicles, the efficiency of neurotransmitter release appears to be comparable between zebrafish hair cells and mouse IHCs (Olt *et al*. 2014). Other similarities between the hair cell ribbon synapses in anamniotes and mammals include the presence of multiple releasable vesicle pools (e.g. zebrafish: Lv *et al*. 2016; frog: Lenzi *et al*. 1999, Graydon *et al*. 2011; mouse: Johnson *et al*. 2005; gerbil: Johnson *et al*. 2017), although-their *in vivo* characteristics are still largely unknown. More recently, fluorescence microscopy has been used to monitor glutamate release from lateral line hair cells during mechanical stimulation (Pichler & Lagnado, 2019; Pichler & Lagnado 2020), which has enabled the investigation of their transfer characteristics *in vivo*. Despite these advances in the understanding of ribbon synapse function, we still have a limited knowledge of the mechanisms leading to sensory adaptation *in vivo*.

We use two-photon Ca^2+^ and glutamate imaging and patch-clamp electrophysiology to investigate sensory adaptation in the zebrafish posterior lateral line system. Hair cell and afferent neuron activity was investigated *in vivo* in response to hydrodynamic stimulations of the mechanoelectrical transduction apparatus using a piezo-driven fluid jet (e.g. Corns *et al*. 2014, 2016; Erickson *et al*. 2017). Prolonged constant displacement of the hair cell stereociliary bundles caused release of glutamate from their ribbon synapses with a biphasic time course. Continuous and repeated stimulation led to synaptic adaptation with a fast presynaptic component of depression, likely due to vesicle depletion, which did not preclude the ability of afferent neurons to phase-lock to periodical stimuli. Recovery from adaptation was observed in both pre- and postsynaptic responses, although they followed different time courses. We have also investigated a form of synaptic adaptation that occurred during hyperpolarising (“negative”) stimuli (Pichler & Lagnado, 2019) and showed that it is likely to be caused by the properties of the hair cell mechanoelectrical transducer apparatus. Our results and approach provide a better understanding of the release characteristics of ribbon synapses *in vivo* and could be very useful in future investigations of synaptic defects in hair cells.

## Materials and Methods

### Ethics Statement and zebrafish lines

All studies were performed at the University of Sheffield and were licensed by the UK Home Office under the Animals (Scientific Procedures) Act 1986 and approved by the University of Sheffield Ethical Review Committee.

Zebrafish were maintained in the aquarium facility of the University of Sheffield on a 14-10 hour light/dark cycle at 28°C. The following zebrafish strains were used: *Tg(brn3c:Gal4)* (Xiao & Baier, 2007), *Tg(UAS:iGluSnFR)* (Marvin *et al*. 2013, Pichler & Lagnado 2019), *Tg(UAS:GCaMP7a)* (Muto *et al*. 2013) and *Tg(nbt:GCaMP3)* (Bergmann *et al*. 2018). *Tg(UAS:iGluSnFR)* and *Tg(UAS:GCaMP7a)* lines were crossed with the *Tg(brn3c:Gal4)* line to drive expression of the fluorescent reporters in lateral line hair cells. GCaMP7a was expressed in at least 6 hair cells per neuromast, and iGluSnFR was expressed in 1 to 4 hair cells per neuromast.

Zebrafish larvae (<5.2 days post fertilisation, dpf) were anaesthetised in tricaine methansulfonate (MS-222) and pinned onto a Sylgard-coated coverslip placed onto the recording chamber by two pieces of tungsten wire (0.025 mm, Advent, Eynsham, UK). The neuromuscular blocker α-bungarotoxin (125 μM, Tocris Bioscience, Bristol, UK) was injected into the heart of the zebrafish to suppress movement. Larvae were then rinsed with extracellular solution (in mM: 140 NaCl, 2 KCl, 2 CaCl_2_, 1 MgCl_2_, and 10 HEPES, pH 7.8, ~290 mOsm) and allowed to recover. Experiments were conducted at room temperature (~21°C).

### Extracellular recordings

Recordings of action potentials (“spikes”) from the cell bodies of the posterior lateral line ganglion (PLLg) neurons were performed using loose-patch voltage-clamp as previously described (Trapani *et al*. 2011; Olt *et al*. 2016). The posterior lateral line ganglion was viewed using a 25× objective, 1.10 NA (Nikon, Japan). Patch pipettes were pulled from borosilicate glass with filaments (Sutter instrument, Novato, USA) using a Narishige puller (PP-830; Narishige Japan). Pipettes were filled with the above extracellular solution and had a pipette resistance between 8 and 20 MΩ. Voltage clamp recordings were performed with a Multiclamp 700B amplifier (Molecular Devices, USA). Data acquisition was performed using pClamp 10 software with a Digidata 1550B board (Molecular Devices). Recordings were sampled at 20 kHz and were routinely low-pass filtered (2.5 kHz, 8-pole Bessel filter) to enhance the signal-to-noise ratio of extracellular spike recordings. All data were stored on a computer for off-line analysis using pCLAMP (Molecular Devices) and Origin (OriginLab Corp., Northampton, MA, USA). The threshold method implemented in the pCLAMP software was used to detect the timing of spike events.

The afferent response to step and periodic stimulation was quantified in terms of firing rate (spike/s), which was computed by convolving the spike train with a gaussian kernel with σ = 200 ms or 5 ms, depending on the experiment. For afferent recordings during periodic stimulation, we quantified the vector strength, a measure of the phase-locking capability of the neuron. We first measured the phase delay *θ_i_* (in the range 0-2π) of each spike from the onset of the period of the stimulation. The vector strength *r* was calculated as the module of the vector obtained by averaging the vectors corresponding to every spike, projected on the unitary circle:

$$r=\sqrt{{\left(\frac{\underset{i}{\sum^{\text{​}}}\cos\theta_{i}}{N}\right)}^{2}+{\left(\frac{\underset{i}{\sum^{\text{​}}}\sin\theta_{i}}{N}\right)}^{2}}$$

where *N* is the total number of spikes during the stimulation. A vector strength of 1 indicates perfect synchronicity.

### Hair bundle stimulation

The mechanical displacement of the cupula containing the stereociliary bundles of the hair cells was performed using a fluid jet from a pipette driven by a 25 mm diameter piezoelectric disc (Corns *et al*. 2014; Erickson *et al*. 2017; Jeng *et al*. 2021; Carlton *et al*. 2021). The fluid jet pipette tip had a diameter of about 20-30 μm and was positioned at about 100 μm from the cupula. The distance of the pipette tip from the cupula was adjusted to elicit a maximal bundle stimulation, by 5-10 μm deflection of the cupula (Zhang *et al*. 2018). To ensure that the fluid jet pressure remained constant for the duration of the stimulation, as well as that there was no backflow at the offset of a stimulation, we routinely monitored the pressure output of the fluid jet by recording the output of a pressure sensor (PendoTech PRESS-S-000, Cole-Parmer) connected to a custom-made instrumentation amplifier (INA122P, Texas Instruments). Saturating mechanical stimuli were applied as steps or sine wave stimuli.

### Two-photon imaging

Signals were recorded using a two-photon laser-scanning microscope (Bergamo II System, Thorlabs Inc., USA) based on a mode-locked laser system operating at 925 nm, 80-MHz pulse repetition rate, < 100-fs pulse width (Mai Tai HP DeepSee, Spectra-Physics, USA) (Ceriani *et al*. 2019). Images were captured by a 60× objective, 1.1 NA (LUMFLN60XW, Olympus, Japan) using a GaAsp PMT (Hamamatsu) coupled with a 525/40 bandbass filter (FF02-525/40-25, Semrock). GCaMP3 signals were recorded at 15 frames per second (fps) with an in-plane resolution of 512 x 512 pixels. To assess responses to short stimuli, a faster frame rate was used to record GCaMP7a signals (117 fps with a frame size of 512 x 128 pixels) and iGluSnFR signals (397 fps with a frame size of 256 x 32 pixels). The frame width ranged between 60 and 90 μm.

For Ca^2+^ and glutamate imaging in hair cells, the focal plane was located close to the basal region of the neuromast, which allowed the basal poles of most of the hair cells to be in focus. For the iGluSnFR recordings, the identification of the optimal focal plane was facilitated by the presence of brighter regions in the hair-cell basolateral membrane, likely indicating the position of a ribbon. Afferent terminals were identified by the presence of well-defined enlargements along the afferent fibres entering the neuromast.

The number of hair cells sampled per neuromast varied between recordings, which was dictated by the number of hair cells expressing the fluorescent reporters and those present in the optimal focal plane. Up to 6 hair cells per neuromast were analysed in fish expressing GCaMP7a and 1-2 cells per neuromast in fish expressing iGluSnFR.

### Imaging analysis

iGluSnFR and Ca^2+^ signals were measured as relative changes of fluorescence intensity (Δ*F/F_0_*), with *ΔF* = *F* – *F_0_*, where *F* is fluorescence at time t and *F_0_* is the average fluorescence of 5 frames at the beginning of the recording (or prior to the stimulus onset). In some experiments, fluorescence traces were filtered using a Savitzky-Golay filter to reduce the noise. Motion correction was routinely performed on two-photon imaging data using the NoRMCorre algorithm of the CaImAn library (Pnevmatikakis and Giovannucci, 2017; Giovannucci *et al*. 2019) using a piecewise-rigid registration. Experiments displaying excessive shift in the focal plane during the recording were manually excluded from analysis. Regions of interest (ROIs) were manually drawn around hair cells and afferent boutons using a custom software. For glutamate and Ca^2+^ imaging measurements from the hair cells, ROIs with bounding box side ranging from 4 to 7 μm were drawn on the synaptic pole. For the analysis of Ca^2+^ imaging data from the hair-cell apical pole, ROIs with bounding box side of ~2 μm were used. For Ca^2+^ imaging in afferent fibres, ROIs with bounding box side of 3 to 7 μm were used. Afferent terminals could be separated from efferent terminals as the latter are unaffected by mechanical stimulation of the neuromast cupula (Pichler & Lagnado 2020).

For iGluSnFR and GCaMP3 experiments, raw fluorescence time-series were corrected for bleaching using this procedure: first, a mono-exponential function *b(t)* was fitted to the raw trace, excluding points where a response to a stimulus was present; secondly, the bleaching-corrected timeseries was calculated as $F_{corr}(t)=\frac{F(t)}{b(t)}\cdot F_{0}$, where *F* is the uncorrected fluorescence at time *t* and *F_0_* is the average fluorescence of 5 frames at the beginning of the recording.

Responses during fast repetitive stimulations (Fig. 4) or periodic stimuli (Fig. 5) were deconvolved using this procedure: 1) first, a mono-exponential function *d*(*t*) = *A* · exp(−*t*/τ) was fitted to the response to a single 50 ms pressure step (time constant: τ = 0.29 s) or to a single cycle of the periodic stimulation; 2) starting from the first stimulation in the train, the *i^th^* peak amplitude *A_i_* of the fluorescence signal was calculated 3) *d(t)* was aligned to the *i^th^* peak and subtracted from the fluorescence trace; 4) steps 2-3 were iteratively repeated for all the stimulations in the train.

Image analysis routines were implemented using Python 3.7 (Python Software Foundation).

### Statistical analysis

Data were tested for normality with the Shapiro-Wilk test with Bonferroni correction for multiple comparisons. Parametric statistics were used for normally distributed data. Data for prolonged stimuli (Baseline, Peak, Steady State) were compared with repeated measurements one-way ANOVA, followed by paired t-test with Bonferroni-adjusted P values for pairwise comparisons. Data for two-step protocols or recovery protocols were compared by repeated measurements two-way ANOVA, followed by one-way models for each significant factor or paired *t*-test. Statistical tests were performed using R (R foundation: R Core Team 2020). Data are displayed as mean ± S.D.

## Results

### Time course of firing rate adaptation and synaptic responses in lateral line hair cells

Synaptic function in hair cells from the zebrafish was investigated from the primary neuromasts (L1–L6) of the posterior lateral line, originating from the first primordium (primI) (Pujol-Martí & López-Schier, 2013).

**Figure 1:**
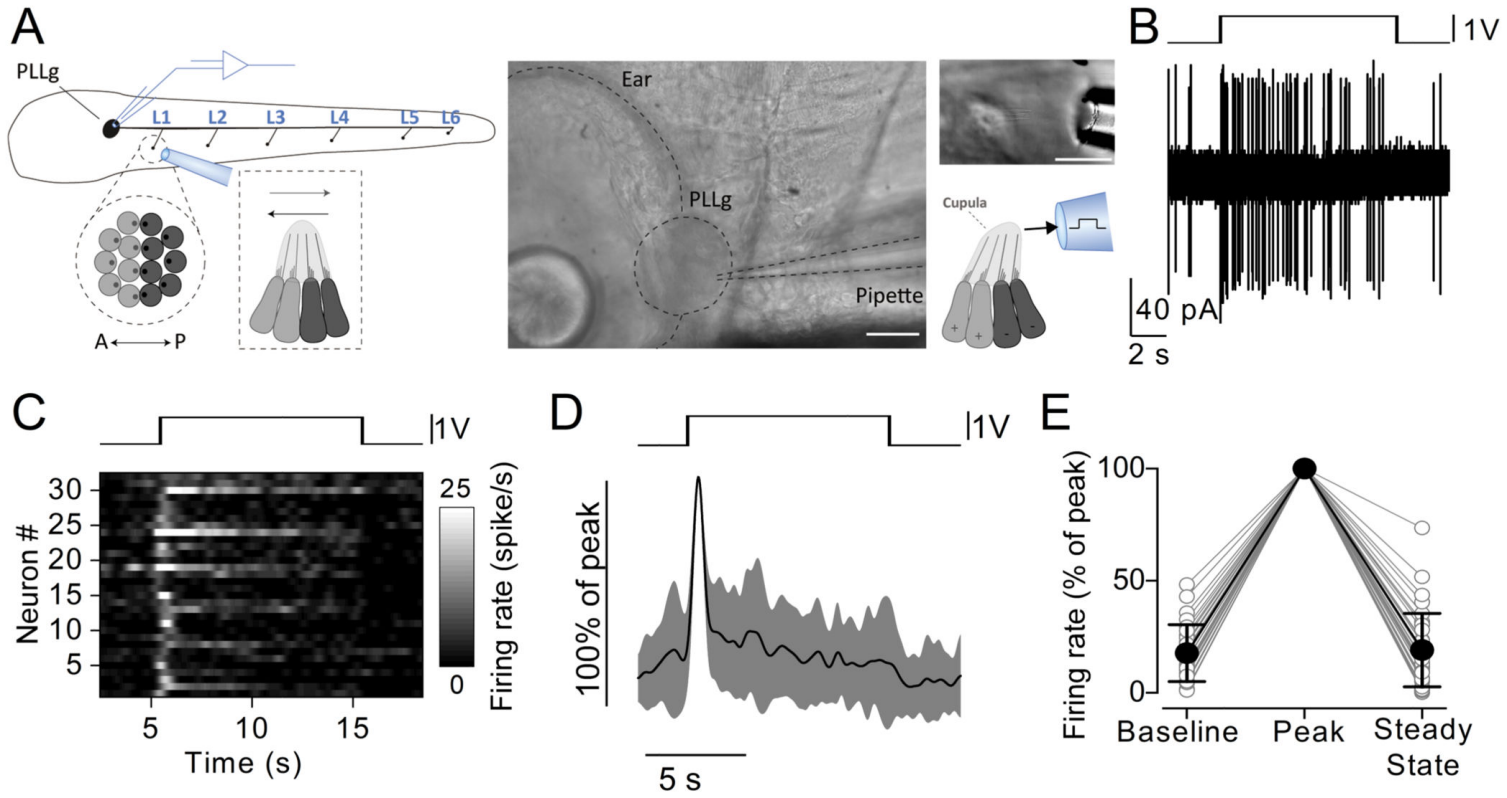
Firing rate adaptation in the afferent neurons during stimulation of individual neuromasts ***A***, Left: schematic overview of the experiment showing the tip of the fluid jet pipette stimulating the zebrafish neuromast L1 while performing loose-patch electrophysiological recordings from the posterior lateral line ganglion (PLLg). Centre: bright-field image of the PLLg, with the recording patch clamp pipette highlighted. Scale bar: 30 μm. Right: piezo-driven fluid jet displacing the cupula of a neuromast, which contains the hair bundles of the hair cells, with saturating stimuli along the anteroposterior axis of the zebrafish. Scale bar: 30 μm. ***B***, Action potential activity from a PLLg afferent neuron while displacing the cupula of a connected neuromast with a 10 s step stimulus (drive voltage to the piezoelectric actuator is shown above the trace). Note the increased firing rate at the onset of the excitatory stimulus and its subsequent adaptation. ***C***, Raster plot of individual afferent neuron activity during the excitatory displacement of the cupula (top: driving voltage, V). The firing rate is normalised to the peak of the firing activity (32 neurons, 29 zebrafish) and was calculated by convolving the spike train with a gaussian kernel (σ = 200 ms). ***D***, Average normalised firing rate of the 32 recordings (solid trace: mean; shaded area: S.D.) during the step displacement (top). ***E***, Average normalised firing rate values. Open circles represent individual recordings. Baseline: average firing rate over at least 30 s before the stimulation. Peak: maximum firing rate in the first 2 s of the stimulus. Steady State: average firing rate during the last 5 s of the stimulus. The baseline firing rate ranged from 0.1 spikes/s to 4.8 spikes/s (median: 1.8 spikes/s, mean: 2.2 spikes/s, 17.7 ± 12.6 % of the peak), while the peak ranged from 4.7 spikes/s to 56.7 spikes/s (median: 12.1 spikes/s, mean: 16.1 spikes/s). The steady state firing rate ranged from 0.0 spikes/s to 9.2 spikes/s (median: 1.9 spikes/s, mean: 2.5 spikes/s, 19.0 ± 16.4% of the peak; *P* < 0.0001, one-way ANOVA, 32 neurons, 29 zebrafish). Pairwise comparison revealed the peak was significantly different from the baseline (*P* < 0.0001) and from the steady state (*P* < 0.0001), while the steady state was not significantly different from the baseline (*P* > 0.9999, paired t tests with Bonferroni-adjusted P values).

To investigate the time-course of adaptation in the posterior lateral line system, we first performed either electrophysiological recordings, Ca^2+^ imaging or glutamate imaging while stimulating individual neuromasts with saturating pressure stimuli using the fluid jet. We used prolonged (10 s long) step stimuli, which allowed us to monitor the full dynamics of pre- and postsynaptic activity (see below). Loose-patch electrophysiological recordings were used to monitor the activity of the posterior lateral line ganglion (PLLg) neurons during stimulation of a single neuromast (Fig. 1A, e.g. Olt *et al*. 2016). In the absence of hair cell stimulation, glutamate is released spontaneously onto afferent terminals generating a resting spiking activity in afferent neurons (Trapani & Nicolson, 2011; Haehnel-Taguchi *et al*. 2014; Levi *et al*. 2015; Olt *et al*. 2016). Consistently with previous observations, spiking activity rapidly increased at stimulus onset (Fig. 1B-E) and subsequently adapted to a lower steady state that was not significantly different from the baseline firing rate (Fig. 1B-E).

**Figure 2:**
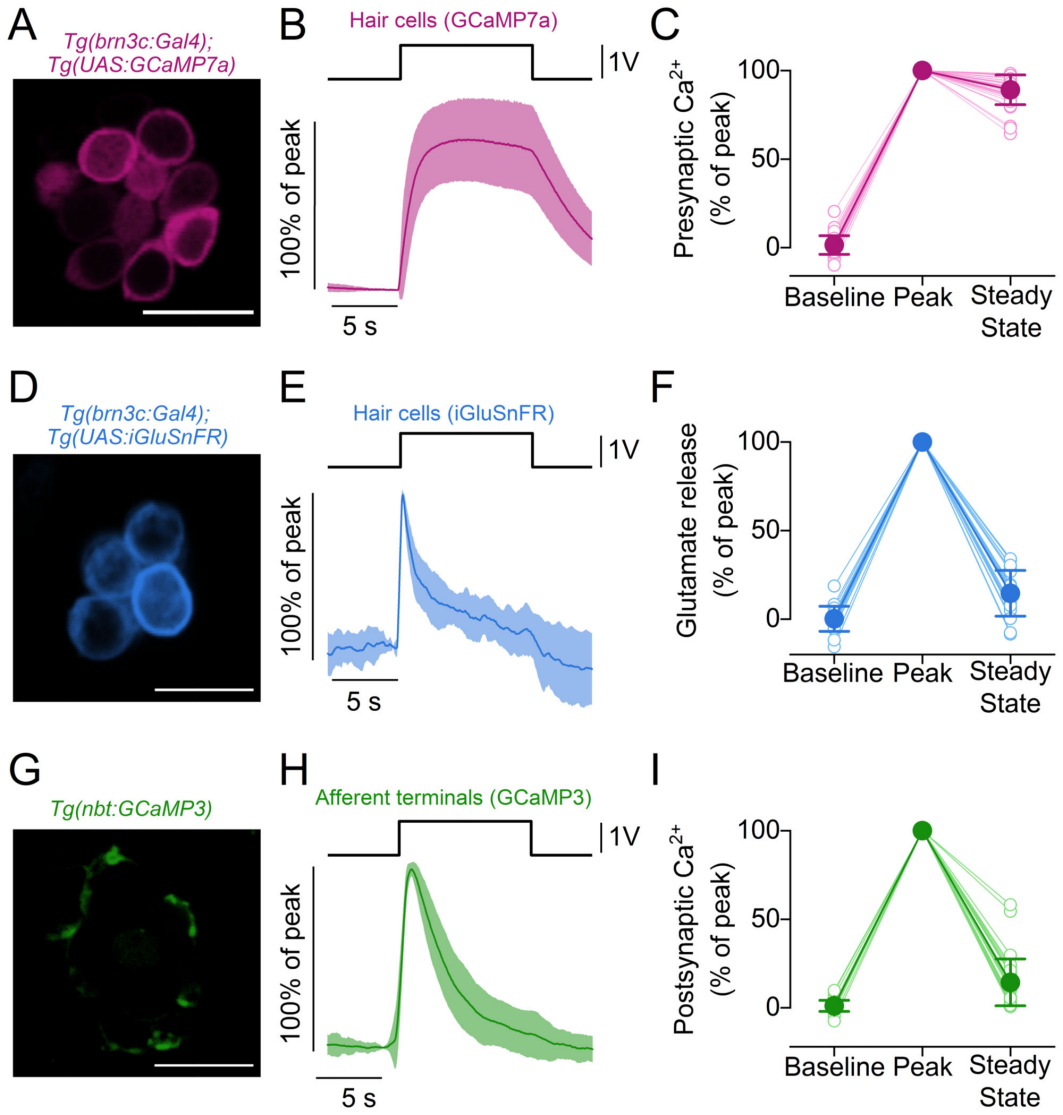
Synaptic responses to prolonged step stimuli in lateral line hair cells ***A***, Maximal projection image of a neuromast expressing the fluorescent Ca^2+^ reporter GCaMP7a in hair cells. Scale bar: 10 μm. ***B***, Average Ca^2+^ changes in hair cells measured as changes in GCaMP7a fluorescence emission. Hair cell bundles were deflected by a 10 s saturating stimulus in the excitatory direction. ***C***, Presynaptic Ca^2+^ changes normalised to the maximum GCaMP7a *ΔF/F_0_* obtained during the first 2 s of the stimulus (Peak). Baseline: average *ΔF/F_0_* before stimulation. Steady State: average *ΔF/F_0_* in the last 5 s of the stimulus. 39 hair cells, 18 neuromasts, 4 zebrafish. ***D***, Maximal projection image of a neuromast expressing the fluorescent glutamate reporter iGluSnFR in hair cells. Scale bar: 10 μm. ***E***, Average traces displaying the time course of glutamate release from the hair cells detected by iGluSnFR during the 10 s stimulus. ***F***, Glutamate release normalised to the Peak of the responses. Baseline, Steady State and Peak are computed as in panel ***B***. 21 hair cells, 15 neuromasts, 7 zebrafish. ***G***, Maximal projection image of a neuromast expressing the fluorescent Ca^2+^ reporter GCaMP3 in postsynaptic terminals. Scale bar: 10 μm. ***H***, Average postsynaptic Ca^2+^ responses measured as changes in GCaMP3 fluorescence during the excitatory bundle displacement. ***I***, Postsynaptic Ca^2+^ responses normalised to the Peak of the response. Baseline, Steady State and Peak are computed as in panel ***C***. 33 afferent terminals, 17 neuromasts, 5 zebrafish. In panels ***B***, ***E*** and ***H***, solid traces represent the mean values and the shaded area the S.D. Open symbols in panels ***C***, ***F*** and ***I*** represent individual recordings.

**Figure 3:**
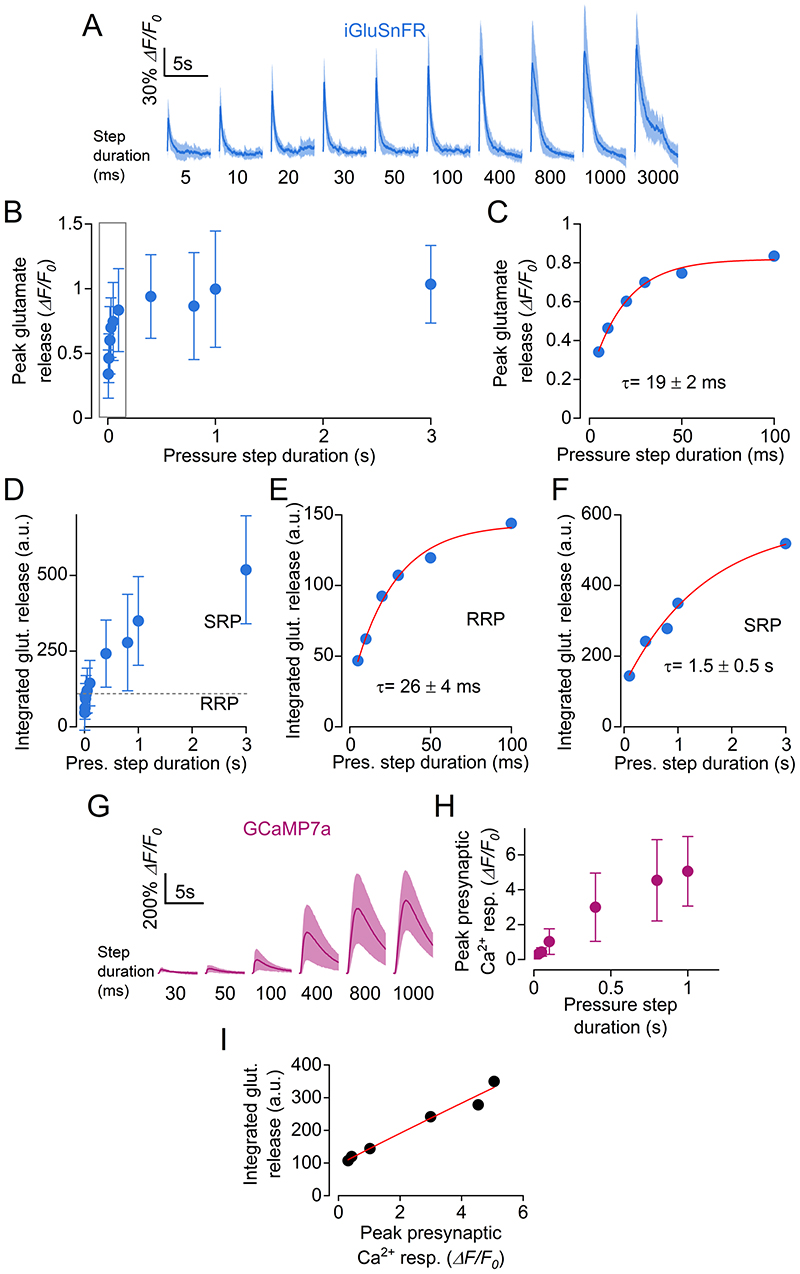
The kinetics of glutamate release indicate the presence of two vesicle pools in hair cells ***A***, Average traces displaying the time course of glutamate release in hair cells detected as changes in iGluSnFR fluorescence emission during fluid jet stimulation with steps of increasing duration, which is indicated below each trace. Number of hair cells from left to right: 14, 15, 14, 13, 16, 16, 16, 17,17, 11 (18 neuromasts; 10 zebrafish). Solid line: mean data; shaded area: S.D.. ***B***, Peak glutamate release as a function of step duration. **C**, Expanded view of peak glutamate release from panel ***B*** (first 100 ms). The peak glutamate release was fitted by a single exponential with τ = 19 ± 2 ms. ***D***, Time integral of iGluSnFR fluorescence traces, indicating the total glutamate release as a function of step duration. ***E, F***, Expanded view of integrated glutamate release for step durations up to 100 ms (***E***, exponential fit: τ = 26 ± 4 ms) and between 200 ms and 3000 ms (***F***, exponential fit: τ = 1.5 ± 0.5 s). ***G***, Average GCaMP7a responses in hair cells to stimuli of indicated duration. Number of hair cells from left to right: 10, 14, 15, 15, 15, 15 (18 hair cells, 13 neuromasts, 5 zebrafish). Solid line: mean data; shaded area: S.D. ***H***, Peak presynaptic Ca^2+^ response as a function of step duration. ***I***, Integrated glutamate release as a function of peak Ca^2+^ response. Pooled data from panels ***F*** and ***H***. Data were fitted with equation: *y = A · x^n^ + C* returned a coefficient *n* = 0.967 ± 0.255, indicating a quasi-linear dependence between neurotransmitter release and Ca^2+^ influx. For the individual recordings used to calculate the averages shown in panels ***B-F, H*** and ***I***, see Supplementary Data Set.

We tested the hypothesis that the spike-rate adaptation was mainly determined by release dynamics at the hair cell synapse using zebrafish expressing different reporters of synaptic activity (Fig. 2). Calcium dynamics in hair cells was determined using zebrafish expressing the Ca^2+^ indicator GCaMP7a (Fig. 2A). We found that the amplitude of presynaptic Ca^2+^ responses remained stable for the duration of the 10 s cupula displacement (Fig. 2B,C), with steady state values displaying only a slight reduction compared to the peak (89.1 ± 8.3 % of the peak, *P* < 0.0001, paired t-test, Bonferroni-adjusted P values, 39 hair cells, Fig. 2C). The lower steady-state values could reflect partial adaptation of the MET current and/or the small and slow inactivation of L-type Ca^2+^ channels (Goutman & Glowatzki, 2007; Olt *et al*. 2014; Zampini *et al*. 2010). We then used the glutamate sensor iGluSnFR (Marvin *et al*. 2013) expressed in lateral line hair cells to monitor neurotransmitter release from the hair cell ribbon synapses, while stimulating the hair cells with the same 10 s step stimuli. Glutamate release rapidly increased at the stimulus onset, reaching a peak within the first ~100 ms (Peak), but quickly adapted to a lower steady state (14.6 ± 12.9 % of the peak, *P* < 0.0001, paired t-test, Bonferroni-adjusted P values, 21 hair cells, Fig. 2D-F). Based on the quasi-linear relationship between presynaptic Ca^2+^ and glutamate release (see Fig. 3I below), this reduction is unlikely to be caused by the slight reduction in the presynaptic Ca^2+^ (~11%, Fig. 2C), but could instead reflect the presence of different pools of synaptic vesicles with distinct release speeds.

Glutamate release from the hair cells triggers Ca^2+^ entry in the afferent terminals, which is thought to be largely due to the activation of Ca^2+^-permeable AMPA receptors (Sebe *et al*. 2017). We used zebrafish expressing GCaMP3 panneuronally (Fig. 2G) to monitor the Ca^2+^ signals in the afferent neuron terminals during the 10 s deflection of the cupula. We found that, similar to presynaptic glutamate release (Fig. 2E), postsynaptic Ca^2+^ responses strongly adapted following an initial rise at the stimulus onset (14.3 ± 13.3 % of the peak, *P* < 0.0001, paired t-test, Bonferroni-adjusted P values, 33 afferent terminals, Fig. 2H,I). These data suggest the presence of a fast depression in the postsynaptic response during a persistent stimulus at the hair cells, which mirrors the depression of glutamate release (Fig. 2E,F).

The above data indicate that the time course of glutamate release at hair cell ribbon synapses is likely to be the main mechanism underpinning sensory adaptation to prolonged stimuli in the lateral line system.

### Vesicle pool dynamics in lateral line hair cells

To investigate the dynamics of vesicle pool release in hair cells, we took advantage of the fact that distinct vesicle pools can be sequentially released by varying the duration of the stimulation, with steps of a few tens of millisecond eliciting the RRP and longer steps additionally recruiting the SRP (Moser & Beutner, 2000; Beutner & Moser, 2001; Johnson *et al*. 2005; Johnson *et al*. 2017).

By performing ultrafast (397 frames per second) two-photon imaging of glutamate release from the hair cells of zebrafish larvae, we were able to detect exocytotic responses during excitatory bundle deflection as short as 5 ms (Fig. 3A). A rapid component of vesicle release was evident at the onset of bundle deflection (Fig. 3A), the peak amplitude of which reached a maximum within the first 100 ms and with a time constant of 19 ± 2 ms (Fig. 3B,C). This rapid time constant is reminiscent of the kinetics of release of the RRP of synaptic vesicles observed in the mammalian IHCs, as reported by measurements of the change in cell membrane capacitance after 50-100 ms voltage steps (e.g. Moser & Beutner, 2000; Beutner & Moser, 2001; Johnson *et al*. 2005; Johnson *et al*. 2017). For steps longer than 100 ms, glutamate release appeared to markedly depress to a plateau (Fig. 3B), which could indicate the presence of a slower pool of synaptic vesicles that can sustain continuous exocytosis after the exhaustion of the RRP. We then calculated the time integral of the iGluSnFR fluorescence responses, since it is more easily comparable with the change in membrane capacitance used to investigate exocytosis in IHCs. The integrated iGluSnFR responses elicited with short (<100 ms) bundle deflections could be approximated with a single exponential of 26 ± 4 ms (Fig. 3D,E), which was comparable to that measured from the peak amplitude (Fig. 3C). Responses to longer stimulations had a much slower time constant (1.5 ± 0.5 s, Fig. 3F), suggesting the presence of a slower secondary pool of synaptic vesicles (SRP: Fig. 3D-F; von Gersdorff *et al*. 1996; Moser & Beutner, 2000; Johnson *et al*. 2005).

In order to measure the Ca^2+^-dependence of glutamate release, we monitored the hair cell Ca^2+^ increase using fast imaging (117 fps) of GCaMP7a fluorescence during excitatory bundle deflections of at least 30 ms (Fig. 3G). By plotting the average integral of glutamate release (Fig. 3D) as a function of average Ca^2+^ responses (Fig. 3H) for bundle displacements between 30 ms and 1000 ms in duration, we found a quasi-linear dependence between neurotransmitter release and hair cell Ca^2+^ increase (Fig. 3I), as previously shown in adult bullfrog hair cells (Keen & Hudspeth, 2006) and mouse IHCs (Johnson *et al*. 2005; Johnson *et al*. 2010; Johnson *et al*. 2017).

### Kinetics of RRP recovery from depletion in lateral line hair cells

**Figure 4:**
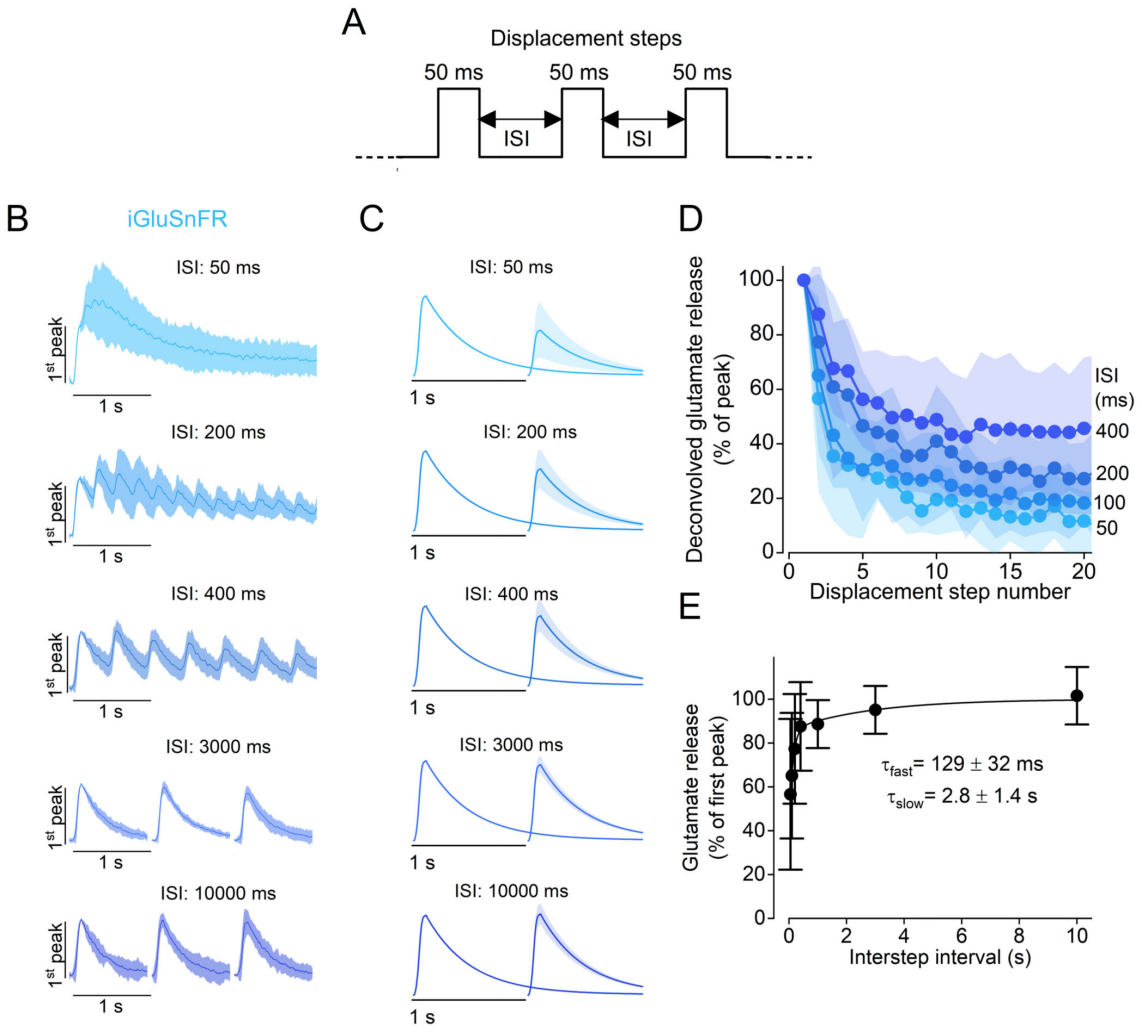
RRP depletion and replenishment in hair cells ***A***, Schematic representation of the stimulus protocol used to displace the cupula of the neuromasts towards the excitatory direction. Step displacements of 50 ms in duration, which saturated the fast glutamate response, were delivered with varying interstep intervals (ISIs). ***B***, Average iGluSnFR responses to a train of steps with different ISIs. Traces are normalised to the peak response of the first step. Note that for longer ISIs (3000 ms and 10000 ms) image acquisition was interrupted in-between steps to limit photobleaching. ***C***, Deconvolved glutamate responses to paired pulses for different ISIs (see Materials and Methods). Individual traces were normalised to the amplitude of the response elicited by the first displacement step. In panels ***B*** and ***C***: solid lines indicate mean value and shaded area the S.D. ***D***, Glutamate release (normalised peak) plotted as a function of pressure step number for the ISIs indicated on the right. ***E***, Time course of RRP replenishment. Peak glutamate release measured at the second displacement step as a function of ISI. The individual data points were fitted with a double exponential function: $y=y_{0}+A_{1}\cdot (1-e^{-\frac{x}{\tau_{fast}}})+A_{2}\cdot (1-e^{-\frac{x}{\tau_{slow}}})$, with *A*_2_ = (1 − *y*_0_ − *A*_1_). Number of hair cells: 10 (50 and 100 ms), 11 (400 ms), 12 (200 ms, 1 and 10 s), 15 (3 s). 33 neuromasts from 16 zebrafish. For the individual recordings used to calculate the averages shown in panels ***D*** and ***E***, see Supplementary Data Set.

We then investigated whether the kinetics of recovery from depletion of the RRP, which is associated with the transient component of glutamate release, allow hair cells to continuously signal during repeated stimulations. Neurotransmitter release from hair cells was measured using iGluSnFR while delivering trains of 50 ms-long step deflections to the cupula, a duration that saturates the fast synaptic response (Fig. 3C,E). When a train of step displacements was delivered with short interstep intervals (ISIs: Fig. 4A), we observed a marked depression of synaptic responses (Fig. 4B-D). For ISIs between 50 and 400 ms, the peak of the glutamate release at each step (Fig. 4D; see Materials and Methods for deconvolution algorithm) reached a plateau within the first 20 steps, suggesting that hair cells can sustain prolonged synaptic release following repeated high-rate stimulation, albeit at a reduced rate of release.

To measure the time-course of recovery of the fast release component from depletion, we measured the peak amplitude of the glutamate response for a pair of pressure steps (50 ms in duration) for ISIs up to 30 s. We found that the recovery of the transient component of glutamate release had a biphasic dependence on the ISI (Fig. 4E), similar to the recovery of the RRP measured in mammalian IHCs (Moser & Beutner, 2000). A fit with a double exponential function yielded a fast time constant of 129 ± 32 ms and a slow time constant of 2.8 ± 1.4 s, with the extrapolated initial level of depression being 42 ± 5% of the initial peak response. These results indicate that a rapid mode of RRP recovery is present in the hair cells, allowing them to sustain continuous signalling during prolonged high-rate stimulation.

### Adaptation of synaptic responses during sinusoidal stimuli

**Figure 5:**
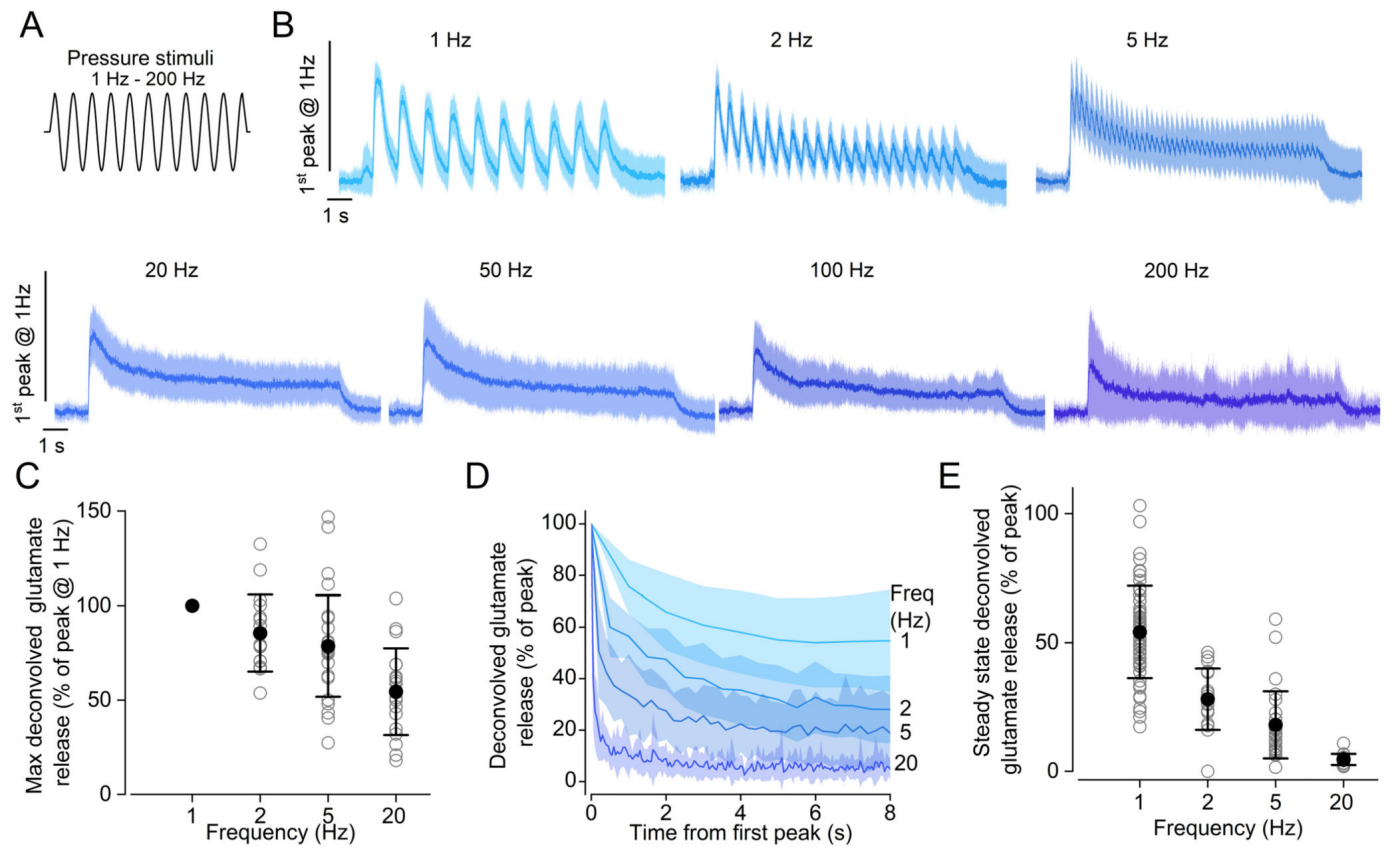
Adaptation of glutamate release in hair cells during periodic stimuli ***A***, Schematic representation showing the 10 s-long sinewave stimulus used to displace the cupula of the neuromasts with the fluid jet. The frequency of the sinewave stimulus used was: 1, 2, 5, 20, 50, 100 and 200 Hz. ***B***, Average iGluSnFR responses (solid line: mean, shaded area: S.D.) recorded in lateral line hair cells using a sine-wave stimulus with the above frequencies. To compare responses to different frequencies, fluorescence traces were normalised to the peak of the response to 1 Hz stimulation. Responses from hair cells with different direction of sensitivity were aligned by shifting the response to one direction by half of the period of the stimulation. ***C***, Maximum glutamate release as a function of the stimulation frequency from 1 Hz to 20 Hz. This analysis was limited to lower frequencies as it was not possible to reliably obtain deconvolved responses from higher frequency stimulations. Responses are normalised to the peak of the response to 1 Hz stimulation. ***D***, Peak glutamate release at each stimulation cycle, as a function of time. The frequency of the stimulus is indicated on the right. Traces are normalised to the peak for each stimulation frequency. The time course of glutamate release could be fitted by a single exponential function at 1 and 2 Hz (1 Hz: τ = 1.4 ± 0.1 s; 2 Hz: τ = 1.0 ± 0.1 s) and a double exponential function at 5 and 20 Hz (5 Hz: τ_fast_ = 0.1 ± 0.01 s, τ_slow_ = 1.5 ± 0.1 s; 20 Hz: τ_fast_ = 0.04 ± 0.01 s, τ_slow_ = 1.0 ± 0.1 s). ***E***, Steady state glutamate release as a function of stimulation frequency. Glutamate responses in panels ***C-E*** were computed by deconvolution of the iGluSnFR fluorescence traces as indicated in Fig. 4 (see also Materials and Methods) and normalised to the maximum response. Number of hair cells: 57 (1 Hz), 17 (2 Hz), 28 (5 Hz), 19 (20 Hz), 21 (50 Hz), 20 (100 Hz), 11 (200 Hz). 56 neuromasts from 18 zebrafish. For the individual recordings used to calculate the averages shown in panels ***D*** and ***E***, see Supplementary Data Set.

The lateral line can encode periodic stimuli up to a frequency of a few hundred Hz (Van Trump & McHenry, 2008) and ganglion neurons are capable of phase-locking to the stimulus (Levi *et al*. 2015). To understand how the kinetics of release influence the encoding of periodic stimuli, we first recorded glutamate release responses while applying saturating sine wave stimulations of different frequency using a fluid jet (1-200 Hz, 10 s-long, Fig. 5A,B). While all hair cells tested showed glutamate release for frequencies between 1 Hz and 20 Hz, a subset did not exhibit detectable iGluSnFR responses for periodic stimuli ≥50 Hz (50 Hz: 1 cell out of 22; 100 Hz: 2 out of 22; 200 Hz: 10 out of 21). For all frequencies tested, the iGluSnFR fluorescence emission rapidly adapted during the 10 s-long stimulation (Fig. 5B). To quantify the degree of adaptation, we used a deconvolution algorithm similar to the one described above (Fig. 4, see Materials and Methods), limiting our analysis to frequencies up to 20 Hz, where the imaging sampling rate allowed to reliably identify the response at each stimulation cycle. We found that the peak glutamate release was maximal at 1 Hz and decreased at higher frequencies, (*P* < 0.0001, one-way ANOVA Fig. 5C). Moreover, synaptic response adaptation was faster and more pronounced at higher frequencies (*P* < 0.0001, one-way ANOVA Fig. 5D,E).

**Figure 6:**
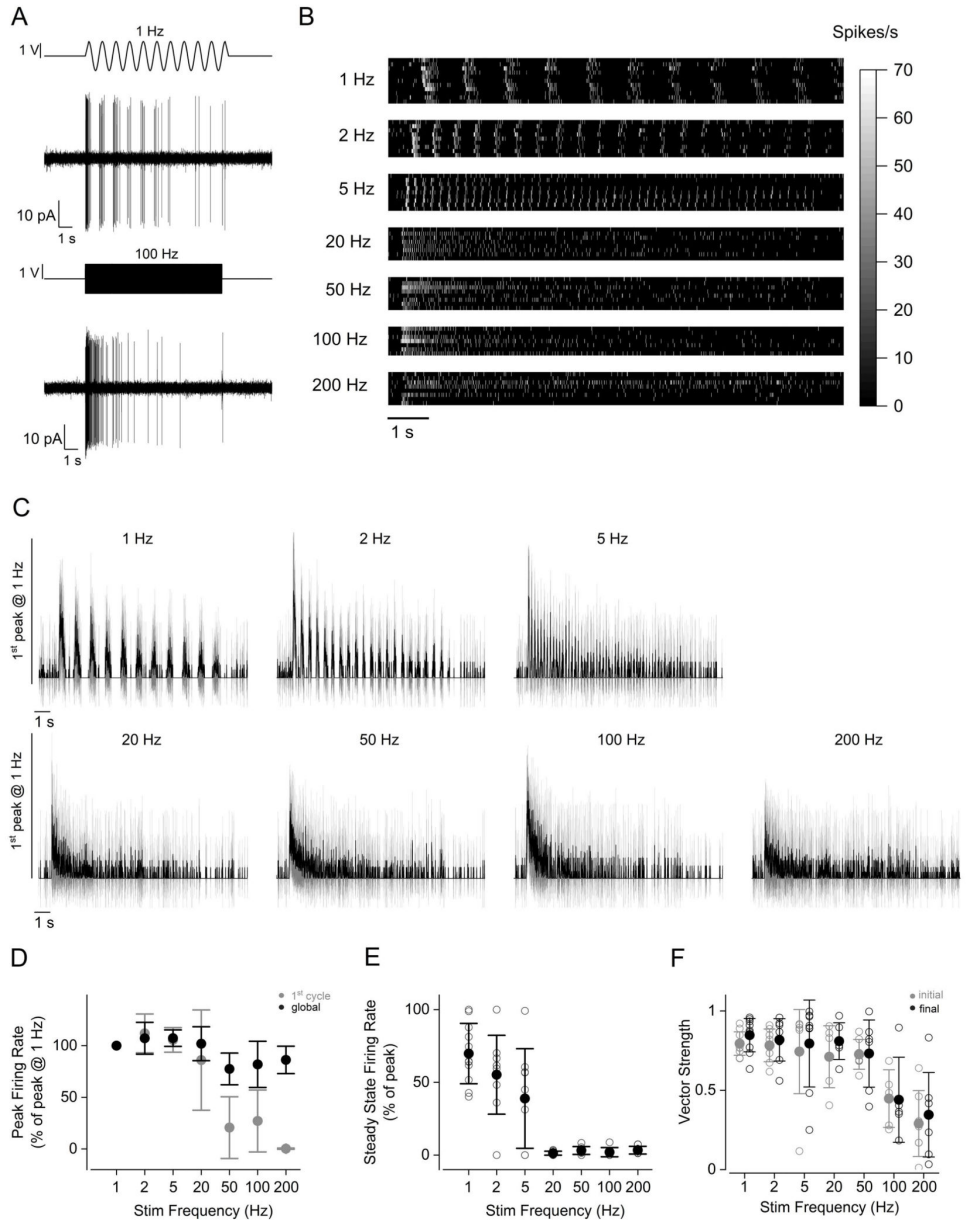
Firing rate adaptation in PLLg neurons during periodic stimuli ***A***, Representative recordings of firing activity from a PLLg neuron during the stimulation of the cupula with a sinewave at 1 Hz (top) and 100 Hz (bottom). ***B***, Raster plot of individual afferent neuron activity during the application of 10 s stimuli. The stimulation frequency is indicated on the left. The firing rate was calculated by convolving the spike train with a gaussian kernel (σ = 5 ms). Of the 11 afferent neuron recordings, 1 showed no firing activity after the initial peak for all the frequencies tested. ***C***, Average firing rate (solid trace: mean; shaded area: S.D.) of PLLg afferent neurons during the application of periodic stimuli in the excitatory direction to a connected neuromast. Traces are normalised to the peak of the response to 1 Hz. ***D***, Peak firing rate during the first excitatory half cycle (grey symbols) and during the entire stimulation (black symbols). Responses are normalised to the peak of the response to 1 Hz stimulation. ***E***, Steady state firing rate as a function of stimulation frequency. Responses are normalised to the peak for each stimulation frequency. The steady state was calculated as the peak firing rate during the last stimulation cycle for frequencies below 5 Hz included, and as the average firing rate in the last 500 ms of stimulation for frequencies higher than 5 Hz. ***F***, Vector strength values as a function of stimulation frequency. Values were computed in two 3 s-long time windows at the beginning (grey symbols) and at the end (black symbols) of the stimulation. Note that a subset of the recordings, for which it was not possible to calculate the final vector strength due to the low number of spikes in the last 3 seconds of stimulation, were not included in this analysis. Number of neurons from left to right (excluded neurons in parentheses): 10 (1), 8 (1), 8 (1), 6 (2), 7 (1), 5 (2), 7 (1), from 7 zebrafish. For the individual recordings used to calculate the averages shown in panel ***D***, see Supplementary Data Set

The above results suggest that the release kinetics of hair cells could have a substantial influence on the encoding of frequency information in the lateral line system during continuous stimulation. To address this hypothesis, we performed patch-clamp recordings from the PLLg while stimulating individual neuromasts with sine-wave stimuli (Fig. 6A,B). The firing activity of ganglion neurons showed a degree of adaptation (Fig. 6C,E) that was comparable to the depression of glutamate release from the hair cells (Fig. 5). We note that for stimulation frequencies ≤20 Hz, the peak of the firing rate usually occurred during the first excitatory half-cycle of the sine wave, while for higher frequencies (50 Hz, 100 Hz and 200 Hz), the peak of the firing rate occurred within the first 100 ms of stimulation (Fig. 6D), which agrees with the hair cell release dynamics described above (Figs. 3-4). We quantified the vector strength, which is a measure of synchronicity between stimulus and response, with a value of 0 indicating a completely stochastic firing activity and a value of 1 indicating synchronicity (see Materials and Methods). By measuring the vector strength at the beginning and at the end of the stimulus, we were able to determine whether the reduction in firing activity affected the capability of the lateral line to phase-lock. In agreement with a previous study (Trapani *et al*. 2009), we found no significant difference between the two conditions (*P* = 0.267, two-way ANOVA, Fig. 6F), suggesting that adaptation does not affect the capability of the lateral line to encode frequency information.

### Recovery from synaptic adaptation

**Figure 7:**
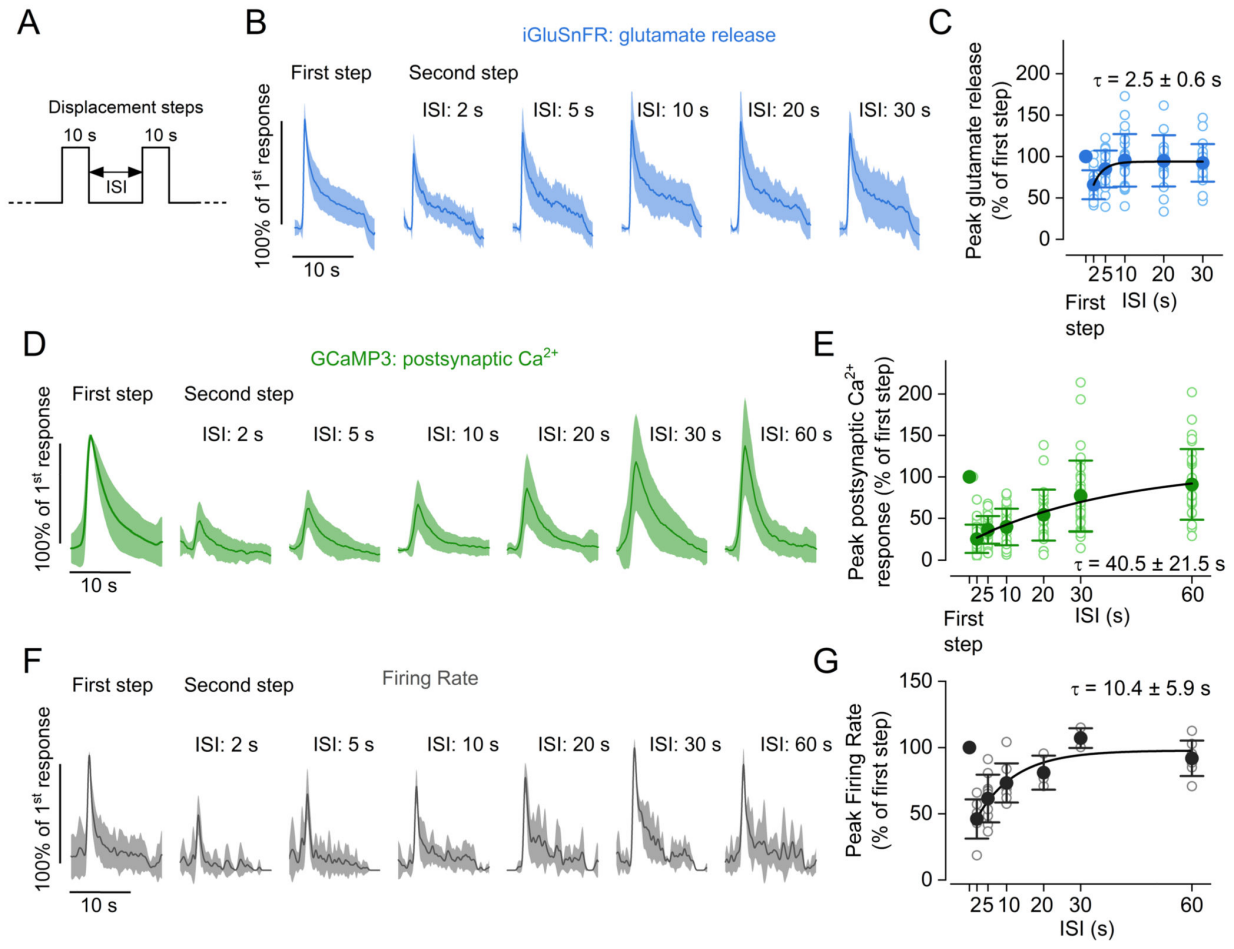
Differential pre- and post-synaptic recovery from adaptation ***A***, Schematic representation of the experimental protocol used to measure the time course of the recovery from adaptation of pre- and postsynaptic responses following two 10 s displacement steps with varying interstep intervals (ISIs). ***B***, Average glutamate release detected as iGluSnFR fluorescence changes in hair cells. ***C***, Peak of response to second step relative to response to first step at different ISIs. The solid line represents an exponential fit to the data. Number of hair cells: 17 (2 and 5 s), 23 (10 s), 16 (20 s), 23 (30 s); 25 neuromasts from 15 zebrafish. ***D***, Average postsynaptic Ca^2+^ responses measured in zebrafish expressing GCaMP3 panneuronally. ***E***, Peak amplitude of response to second step relative to response to first step at different ISIs. Open symbols represent individual recordings and filled symbols denote average values. The solid line represents an exponential fit to the data. Number of afferent terminals: 29 (2 s), 31(5 s), 33 (10 s), 25 (20 s), 35 (30 s), 29 (60 s); 33 neuromasts from 10 zebrafish. ***F***, Average normalised firing rate of afferent neurons. ***G***, Average firing rate during second step relative to first step at different ISIs. The solid line represents an exponential fit to the data. Number of neurons: 7 (2 s), 9 (5 s), 8 (10 s), 3 (20 s), 3 (30 s), 7 (30 s) from 18 zebrafish. In panels ***B, D*** and ***F***, responses are normalised to the maximum amplitude of the first step responses at indicated ISIs. Solid traces represent the mean values and the shaded area the S.D.. In panels ***C***, ***E*** and ***G***, filled and open symbols denote average values and individual recordings, respectively. Solid lines represent a fit to the data with the function $y=y_{0}+A\cdot \left(1-e^{-\frac{x}{\tau }}\right)$.

The time course of recovery from adaptation was measured as the response to a second displacement step that was delivered after a 10 s saturating stimulus (Fig. 7A), which completely depress the synaptic responses (Figs. 1-2). Using zebrafish expressing iGluSnFR, we found that glutamate release from the hair cells recovered with a time constant (peak: τ = 2.5 ± 0.6 s, Fig. 7B,C) compatible to that previously determined for the slow component of the RRP refilling (Fig. 4). The peak glutamate release values during the second step fully recovered within 5-10 s ISIs (2 s: *P* < 0.0001, 5 s: *P* = 0.065, 10 and 20 s: *P* >0.9999, paired *t*-tests with Bonferroni adjusted *P* values). These results suggest that the recovery from synaptic adaptation is largely determined by the replenishment of the RRP. Conversely, we found that postsynaptic Ca^2+^ responses (Fig. 7D) recovered with a slower time constant of τ = 40.5 ± 21.5 s. As a result, the normalised peak of the second response was significantly different from the first at all ISIs up to 30 s (*P* < 0.0001 for all stimuli apart 30 s and 60 s: *P* = 0.018 and *P* > 0.9999, respectively, paired *t*-tests with Bonferroni adjusted *P* values, Fig. 7E). This observation raises the possibility that specific postsynaptic mechanisms are likely to contribute to synaptic depression, providing evidence for a distinct adaptation component at hair cell ribbon synapses.

The recovery of the firing rate of PLLg neurons (Fig. 7F) had an intermediate time course compared to the fast recovery of glutamate release and the slow recovery of postsynaptic Ca^2+^ responses (peak firing rate: τ = 10.4 ± 5.9 s, Fig. 7G; average firing rate: τ = 12.0 ± 6.9 s, data not shown). This measurement suggests that distinct classes of glutamate receptors may contribute differently to synaptic depression at the hair cell synapse. Indeed, both Ca^2+^-permeable and Ca^2+^-impermeable glutamate receptors have been shown to be present at the post-synaptic afferent synapses in hair cells (Sebe *et al*. 2017). We also observe that the readout of postsynaptic Ca^2+^ responses may not represent a precise quantification of synaptic transfer at the hair cell ribbon synapses.

### Adaptation during inhibitory displacements of the MET apparatus

**Figure 8:**
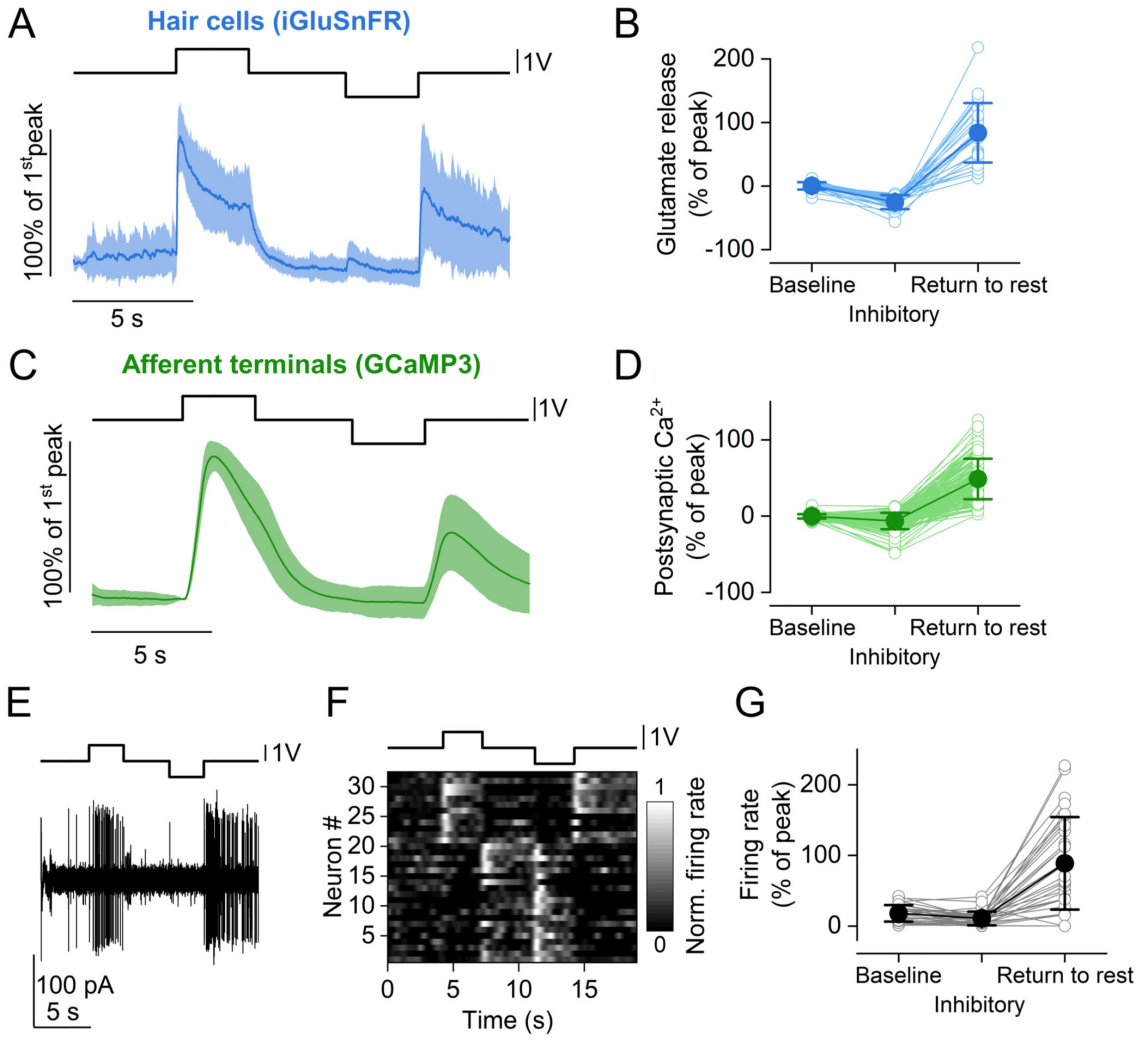
Pre- and postsynaptic responses to inhibitory cupula displacement ***A***, Average time course of iGluSnFR responses to two consecutive saturating stimuli (duration: 3s) in opposite directions (excitatory and inhibitory) from 26 hair cells (23 neuromasts, 15 zebrafish). Top trace: fluid jet driving voltage. ***B***, Glutamate release at three different time points of the stimulus. Values are normalised to the maximum response calculated during the first 2 s of the stimulus (Peak). Baseline : average *ΔF/F_0_* before stimulation. Inhibitory: minimum *ΔF/F_0_* during the inhibitory step. Return to rest: maximum *ΔF/F_o_* in the 3 s after the termination of the inhibitory stimulation. The return to rest response was visible in the majority of hair cells tested (26 out of 28 hair cells). ***C***, Average trace of postsynaptic Ca^2+^ responses to two 3 s saturating stimuli in opposite directions (excitatory and inhibitory) detected as change in GCaMP3 fluorescence emission from 143 afferent terminals (69 neuromasts, 35 zebrafish). ***D***, Postsynaptic Ca^2+^ responses during the delivery of the stimulus. Values are normalised to the maximum response calculated during the first 2 s of the stimulus (Peak). Baseline, Inhibitory and Return to rest were calculated as in panel ***B***. ***E***, Representative electrophysiological recording from one afferent neuron while stimulating a connected neuromast. Note the increase in firing rate both for the positive (excitatory) pressure stimulus and at the return to rest from the negative (inhibitory) stimulus. ***F***, Raster plot of individual afferent neuron activity during the application of two 3 s saturating stimuli in opposite directions. The firing rate is normalised to the peak of the firing activity during the excitatory step and was calculated by convolving the spike train with a gaussian kernel (σ = 200 ms). ***G***, Quantification of PLLg neuron activity during the delivery of the stimulus (32 neurons, 16 zebrafish.). Values are normalised to the maximum response calculated during the first 2 s of the stimulus (Peak). Baseline, Inhibitory and Return to rest were calculated as in panel ***B***. In panel ***A*** and ***C***, solid traces represent mean and shaded areas S.D. In panels ***B***, ***D*** and ***G***, filled symbols denote average values and open symbols represent individual recordings.

We investigated the adaptation properties of lateral line hair cells during inhibitory displacements of the cupula, which deflect the stereociliary bundle in the direction opposite to the hair cell sensitivity and reduce the open probability (*P*_o_) of the MET channels. Glutamate release from the hair cells was found to increase significantly at the offset of inhibitory displacement steps (*P* < 0.0001, paired t-test, Bonferroni-adjusted P values, 28 hair cells, Fig. 8A-B), in agreement with previous observations (Pichler *&* Lagnado, 2019). These responses were mirrored postsynaptically by the increased Ca^2+^ entry in the afferent terminals (*P* < 0.0001 compared to baseline, paired t-test, Bonferroni-adjusted P values, 143 afferent terminals, Fig. 8C,D) and spike rate of the PLLg neurons (*P* < 0.0001 compared to baseline, 32 afferent neurons, Fig. 8E-G) at the offset of inhibitory displacements.

**Figure 9:**
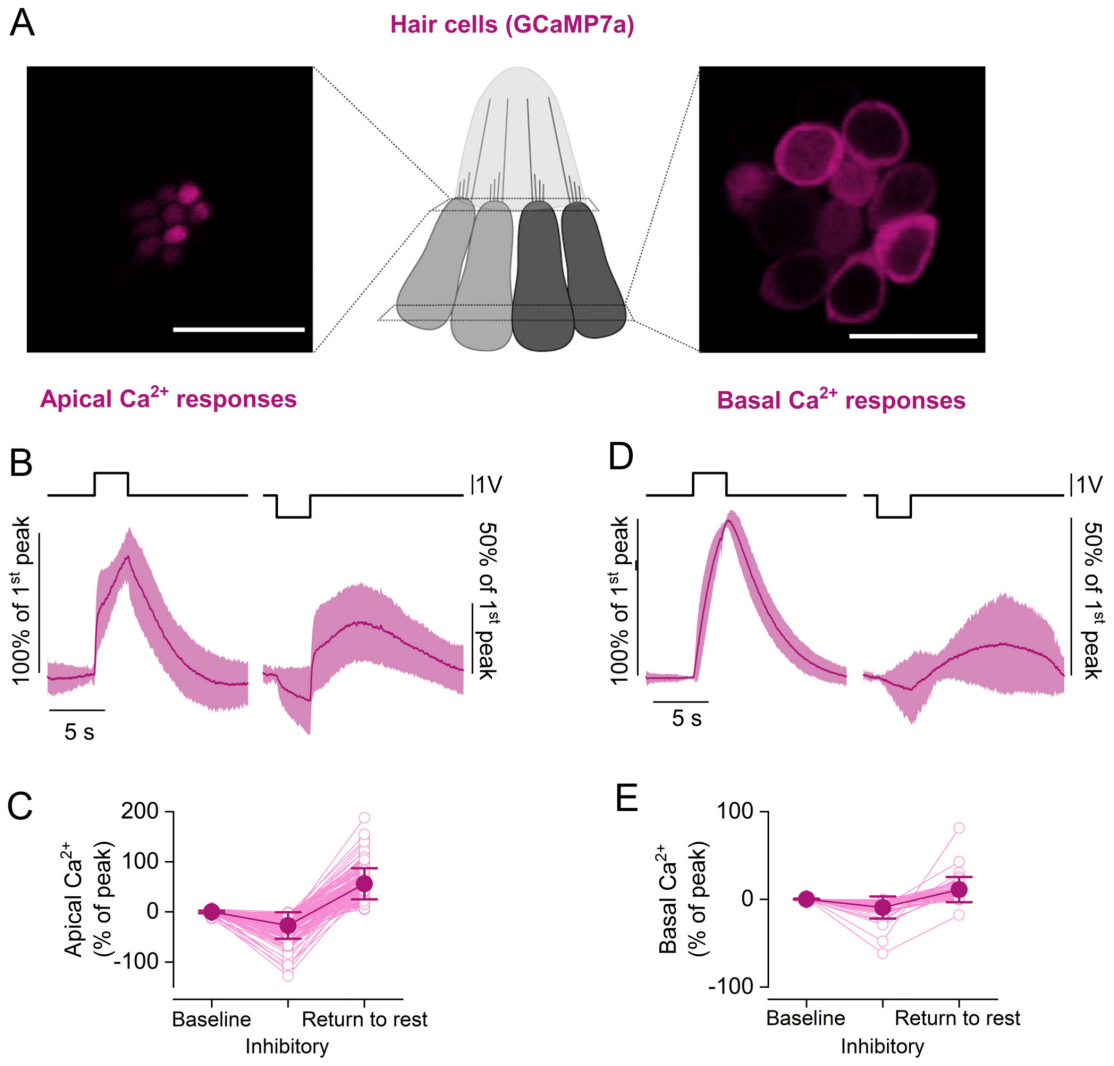
Ca^2+^ responses to inhibitory cupula displacement in apical and basal hair cell compartments ***A***, Maximal projection images of a neuromast expressing the fluorescent Ca^2+^ reporter GCaMP7a in hair cells. Two focal planes are shown: the hair cell apical pole (left), where fluorescence signals largely reflect Ca^2+^ entry through the MET channels; hair cell basal pole (right), where fluorescence signals reflect Ca^2+^ entry through voltage-gated Ca^2+^ channels. Scale bar: 10 μm. ***B***, Average time course of intracellular Ca^2+^ in hair cells measured as change in GCaMP7a fluorescence at the hair cell apical pole (142 hair cells, 28 neuromasts, 8 zebrafish). Two 3 s saturating stimuli in the excitatory (left) and inhibitory (right) directions were delivered. Note the negative deflection of intracellular Ca^2+^ concentration during the negative step, followed by a slow increase above baseline levels upon return to rest. ***C***, Ca^2+^ changes in the hair cell apical pole during the delivery of the excitatory and inhibitory stimuli. Values are normalised to the maximum GCaMP7a signal during the excitatory step. Baseline: average *ΔF/F_0_* before stimulation. Inhibitory: minimum *ΔF/F_0_* during the inhibitory step. Return to rest: maximum of the response in the 12 seconds after the termination of the inhibitory stimulation. The return to rest response was visible in the majority of hair cells tested (142 out of 152 hair cells). ***D***, ***E*** same as in ***B*** and ***C***, but Ca^2+^ responses were measured at the hair cell basal (synaptic) pole (28 hair cells, 9 neuromasts, 3 zebrafish). The inhibitory displacement of the cupula caused a reduction in Ca^2+^ concentration, followed by a positive “rebound” upon returning to the rest position. The return to rest response was visible in 28 out 45 hair cells. The top traces in panels ***B*** and ***D*** represent the fluid jet driving voltage. In panels ***B*** and ***D***, the mean values and the shaded area the S.D. In panels ***C*** and ***E***: open symbols represent individual recordings; filled symbols denote average values.

To investigate whether the release of glutamate during the inhibitory step displacements was linked to opening of the MET channels, we monitored Ca^2+^ dynamics in the apical and basal portion of hair cells using zebrafish expressing GCaMP7a (Fig. 9*A*). At the hair cell apex, which is just below the MET apparatus, Ca^2+^ signals decreased during inhibitory displacements of the cupula, but rapidly increased during excitatory stimuli and upon returning the hair bundles to their resting position (*P* < 0.0001, 152 hair cells, Fig. 9B,C). A slower, positive “return to rest” response following a decrease in GCaMP7a fluorescence during inhibitory stimuli was also observed in the Ca^2+^ signal at the basal pole of the hair cell (*P* < 0.0001, 45 hair cells, Fig. 9D,E). These Ca^2+^ changes mirrored the time-course of glutamate release at the synapses (Fig. 8A,B), indicating that, in the presence of negative stimuli, the lateral line may extend its sensitive range due to a mechanism mediated by the adaptation properties of the MET channels located at the top of the hair-cell stereocilia.

## Discussion

In this study, we investigated the biophysical properties of hair cells *in vivo* in the context of sensory adaptation, using the zebrafish lateral line. Prolonged saturating steps in the excitatory direction or sinusoidal mechanical stimuli, which caused maximal deflection of the cupula and the full opening of the MET channels, led to the adaptation of the firing rate of PLLg neurons (Figs. 1,6). The release kinetics of glutamate from the hair cell ribbon synapses (Figs. 2-5) represent the limiting step contributing to postsynaptic adaptation. The time course of glutamate release was characterised by two kinetically distinct components. The initial phasic component was saturated within 50-100 ms and comparable to that of the RRP response in mammalian cochlear IHCs. Although the rapid adaptation of the PLLg neuron firing rate is primarily of presynaptic origin, postsynaptic mechanisms are also likely to contribute as postsynaptic Ca^2+^ responses had a slower recovery time course compared to the recovery of glutamate release (Fig. 7). A distinct adaptation mechanism was also observed at the offset of inhibitory cupula displacements (Fig. 8), which is during the return of MET channels to their resting open probability (Fig. 9), highlighting a shift in the sensitivity range of the lateral line during prolonged stimulation.

### Kinetics of release in lateral line hair cells during hair bundle displacement *in vivo*

Ribbon synapses in hair cells, photoreceptors and retinal bipolar cells allow both fast and sustained release through the exocytosis of pools of vesicles with different release speeds, coupled with highly efficient mechanisms of vesicle replenishment. The pool of membrane-proximal vesicles is thought to represent the morphological correlate of the functionally determined RRP, which mediates the fastest component of exocytosis when measured using membrane capacitance tracking approaches (Parsons *et al*. 1994; von Gersdorff & Matthews, 1996; Moser & Beutner, 2000; Johnson *et al*. 2005; Johnson *et al*. 2017). The time constant of saturation of the RRP release is in the tens of millisecond (e.g. turtle papilla hair cells: Schnee *et al*. 2005; mouse IHCs: Moser & Beutner, 2000; Johnson *et al*. 2005, mouse type I vestibular hair cells: Dulon *et al*. 2009; gerbil IHCs: Johnson *et al*. 2017). These measurements are comparable to those obtained with our optical approach using step or sinusoidal stimulations, showing that the transient component of glutamate release in lateral line hair cells *in vivo* saturated within 50-100 ms (τ = ~20-30 ms). This is about one order of magnitude faster than the time constant previously measured in zebrafish lateral line hair cells using capacitance measurements (Lv *et al*. 2016). After the initial phasic component, lateral line hair cells were capable of sustaining release for the entire duration of the stimulation protocols (Figs. 2,4,5), albeit at reduced rates. This is consistent with the recruitment of a slower pool, as observed in other hair cell ribbon synapses by both capacitance measurements (e.g. Moser & Beutner, 2000; Johnson *et al*. 2005; Spassova *et al*. 2004; Johnson *et al*. 2008, Von Gersdorff & Mathews, 1996) and postsynaptic recordings (e.g. Goutman & Glowatzki, 2007). Overall, our data highlight a high degree of evolutionary conservation of several aspects of hair cell ribbon synapse function in diverse organisms.

Vesicles pools with different kinetics are thought to underlie different computations necessary for coding sensory stimuli in both the auditory (Delgutte, 1980) and visual systems (Oesch & Diamond, 2011). In the lateral line, the fast response is likely to be important for triggering rapid escape responses, a behaviour that relies on the timing of excitatory and inhibitory inputs to reticulospinal neurons (Kohashi & Oda, 2008). On the other hand, continuous release may underlie more sophisticated computations that require integration of information from different neuromasts, such as rheotaxis (Valera *et al*. 2021, Oteiza *et al*. 2017). Behavioural investigation of specific mutants with impaired synaptic release will be required to directly test these hypotheses.

The linear dependence between glutamate release and intracellular Ca^2+^ is reminiscent of the relationship between exocytosis and Ca^2+^ current found in mature mouse IHCs (Johnson *et al*. 2005; 2010), although a supra-linear relationship might be masked in our experiments by the use of a cytosolic Ca^2+^ indicator (GCaMP7a), which reports bulk changes in intracellular Ca^2+^ concentration. The use of a synaptically-localised sensor (e.g. Dreosti *et al*. 2011) would be needed to accurately measure the intrinsic Ca^2+^ dependence of release at lateral line hair cell ribbon synapses.

### Adaptation and recovery in the lateral line system

Adaptation to sustained stimuli is a common feature of many sensory neurons, including auditory nerve fibres in both lower vertebrates and mammals (e.g. goldfish: Furukawa & Matsuura, 1978, monkey: Nomoto *et al*. 1963, ferret: Sumner & Palmer, 2012). In the cochlea, it is thought that the adaptation of the firing rate observed in the afferent neurons is primarily due to the partial depletion of the RRP (Goutman & Glowatzki, 2007, Moser & Beutner, 2000, Spassova *et al*. 2004). We showed that a similar adaptation mechanism is likely to be present *in vivo* in lateral line hair cells. Prolonged stimulation of the cupula produced nearly-constant presynaptic Ca^2+^ signals in hair cells, in agreement with the reported lack of Ca^2+^ current inactivation (Olt *et al*. 2014), but caused adaptation of glutamate release, which was mirrored by the postsynaptic Ca^2+^ responses at afferent terminals and firing rate of PLLg neurons. Despite a decrease in postsynaptic responses, the ability of lateral line afferent neurons to phase lock to sinusoidal mechanical stimuli was unaffected. This characteristic of the lateral line is crucially dependent on synaptic vesicle recycling, since phase-locking was reduced during prolonged stimulation in *Synj1* mutant zebrafish, in which vesicle recovery is impaired (Trapani *et al*. 2009). We also found that Ca^2+^ responses in afferent terminals following prolonged step stimulation had a relatively slow recovery time course, requiring at least 60 s to return to the pre-stimulus level. This was significantly slower than the time course of glutamate release under the same stimulation conditions, which recovered to pre-stimulus levels in ~10 seconds and with a time constant compatible with the recovery of the RRP. This suggests that postsynaptic mechanisms contribute to the adaptation of lateral line responses to sustained stimuli.

Calcium entry into afferent terminals occurs via N-methyl-D-aspartate (NMDA) receptors and Ca^2+^-permeable AMPA receptors lacking the GluA2 subunit, the latter being considered the major Ca^2+^ entry route in lateral line afferent terminals (Sebe *et al*. 2017). It is therefore likely that desensitisation of AMPA receptors occurs during prolonged stimulation, similarly to what has been reported in the rat afferent spiral ganglion neurons innervating the IHCs (Goutman & Glowatzki, 2007; Goutman, 2017) and at bullfrog amphibian papilla hair cell synapses (Graydon *et al*. 2014). Moreover, Ca^2+^-permeable AMPA receptors lacking the GluA2 subunit lose sensitivity following AMPA exposure (Sebe *et al*. 2017) and are implicated in excitotoxicity in the lateral line (Sebe *et al*. 2017) and in the cochlea (Hu *et al*. 2020). As such, desensitisation of a subset of postsynaptic AMPA receptors may exist as a protective mechanism to prevent excitotoxicity.

### “Off” responses in the lateral line system

We showed that the lateral line hair cells are capable of signalling the return to rest of the MET apparatus from an “off” position, i.e. a condition in which the hair bundle is deflected in the inhibitory direction (Figs. 8, 9), which is in agreement with a previous work (Pichler & Lagnado, 2019). Our data also indicate that pre- and postsynaptic responses at the offset of the inhibitory stimuli are likely to be regulated by the adaptation properties of the MET channel. One of the manifestations of the MET channel adaptation is the resting open probability (*P*_o_) (e.g. Eatock *et al*. 1987; Assad *et al*. 1989; Crawford *et al*. 1991; Corns *et al*. 2014; Marcotti *et al*. 2016). Because of this resting *P*_o_, hair bundle stimulation in the inhibitory direction shuts off the fraction of the MET current flowing at rest, yet a large but transient rebound inward MET current is observed at the stimulus offset. We showed that this depolarising MET current rebound, which can be visualised by the rapid increase in Ca^2+^ just below the stereociliary bundles, caused a Ca^2+^ elevation at the hair cell synaptic region responsible for triggering glutamate exocytosis.

We also show that “off” responses are encoded in the firing activity of the primary afferent neurons and therefore are likely to have a role in the computation of sensory information in the brain. Indeed, responses to the cessation of stimulation have been previously reported in flow-responsive neurons in the larval zebrafish brain (Vanwalleghem *et al*. 2020). Neural responses at the offset of a sensory stimulus have been studied in the visual system (Schiller *et al*. 1986) and have been reported in the auditory system, where neurons responding to the cessation of sound are found throughout the auditory pathway (Kopp-Scheinpflug *et al*. 2018). This property of lateral line hair cells may provide the brain with important cues regarding the timing and spectral content of a stimulus and it is likely to be crucial in maintaining the lateral line sensitivity in the presence of sustained stimuli.

## Supplementary Material

Supplementary Figure

## Data Availability

The data that support the findings of this study are available from the corresponding authors upon reasonable request.

## Key Points Summary

- The aim was to determine the sensory adaptation characteristics at hair-cell ribbon synapses *in vivo* using two-photon imaging and electrophysiology.
- Hair cells of the zebrafish lateral line transmit hydrodynamic stimuli to the PLLg afferent neurons.
- Excitatory hair bundle deflections by water-jet stimuli cause glutamate release at the synapses with a rapid (phasic) and a sustained component, likely linked to the exocytosis of the RRP and SRP, respectively.
- Glutamate-induced increase in afferent neuron firing rate adapts over time, which is mirrored by the depression of neurotransmitter release, without preventing phase-locking.
- Adaptation also occurs at the offset of inhibitory hair-bundle displacements, highlighting a shift in the sensitivity range of the lateral line during prolonged stimulation.
- Postsynaptic mechanisms exert some degree of regulation on the afferent firing adaptation.
- We conclude that vesicle depletion is the primary determinant of firing rate adaptation, allowing lateral line hair-cell ribbon synapses to maintain sensitivity to sustained stimuli.
