# Supplementary material for: Sensory adaptation at ribbon synapses in the zebrafish lateral line
Source: J Physiol. Author manuscript; Available in PMC 2021 Dec 23. (PMC7612133; doi:10.1113/JP281646)
Supplement: Supplementary Figure [file EMS140584-supplement-Supplementary_Figure.pdf]

Sensory adaptation at ribbon synapses in the zebrafish lateral line

Francesca De Faveri, Walter Marcotti, Federico Ceriani

Data points for Figure 3B-F,H,I; Figure 4D,E; Figure 5D; Figure 6D;

Figure 3

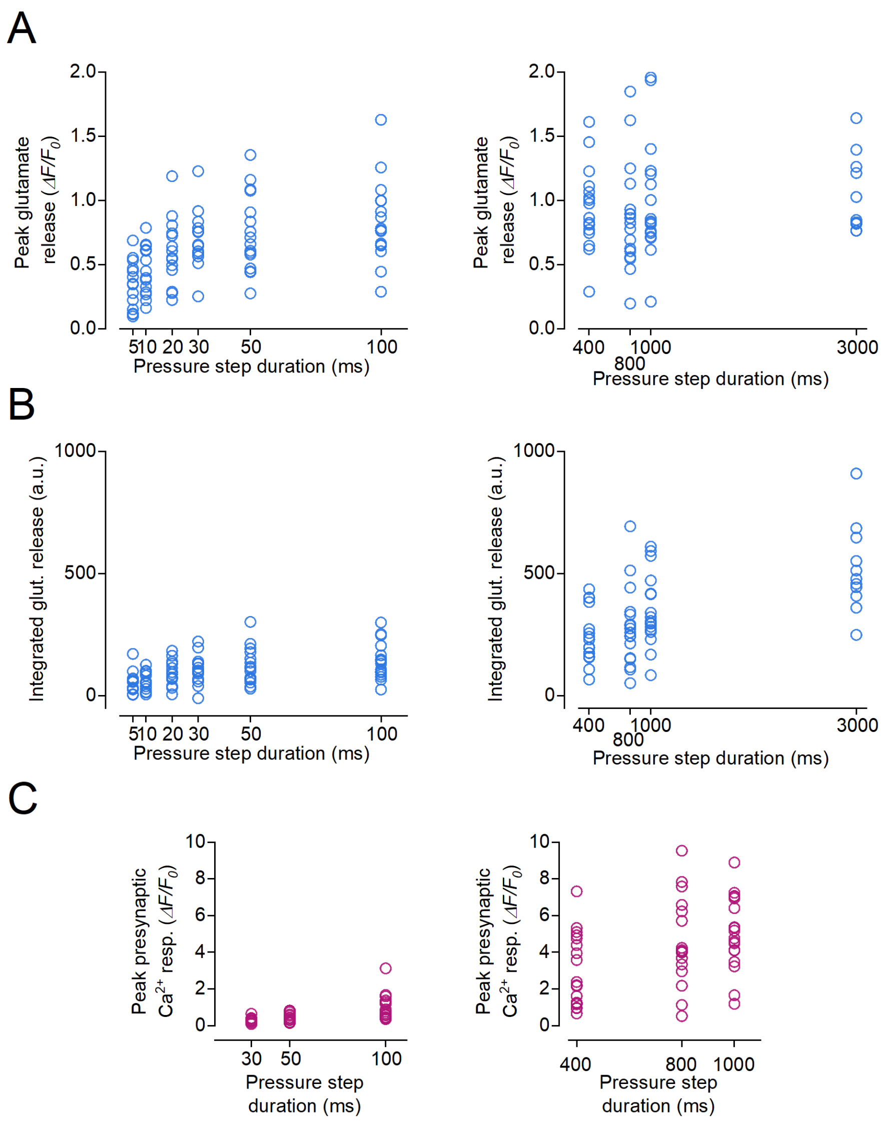

Figure 3. The kinetics of glutamate release indicate the presence of two vesicle pools.

A, Individual datapoints for **Figure 3B,C**. **B**, Individual datapoints for Figure **3D-F,I**. **C**, Individual datapoints for **Figure 3H,I**.

Figure 4

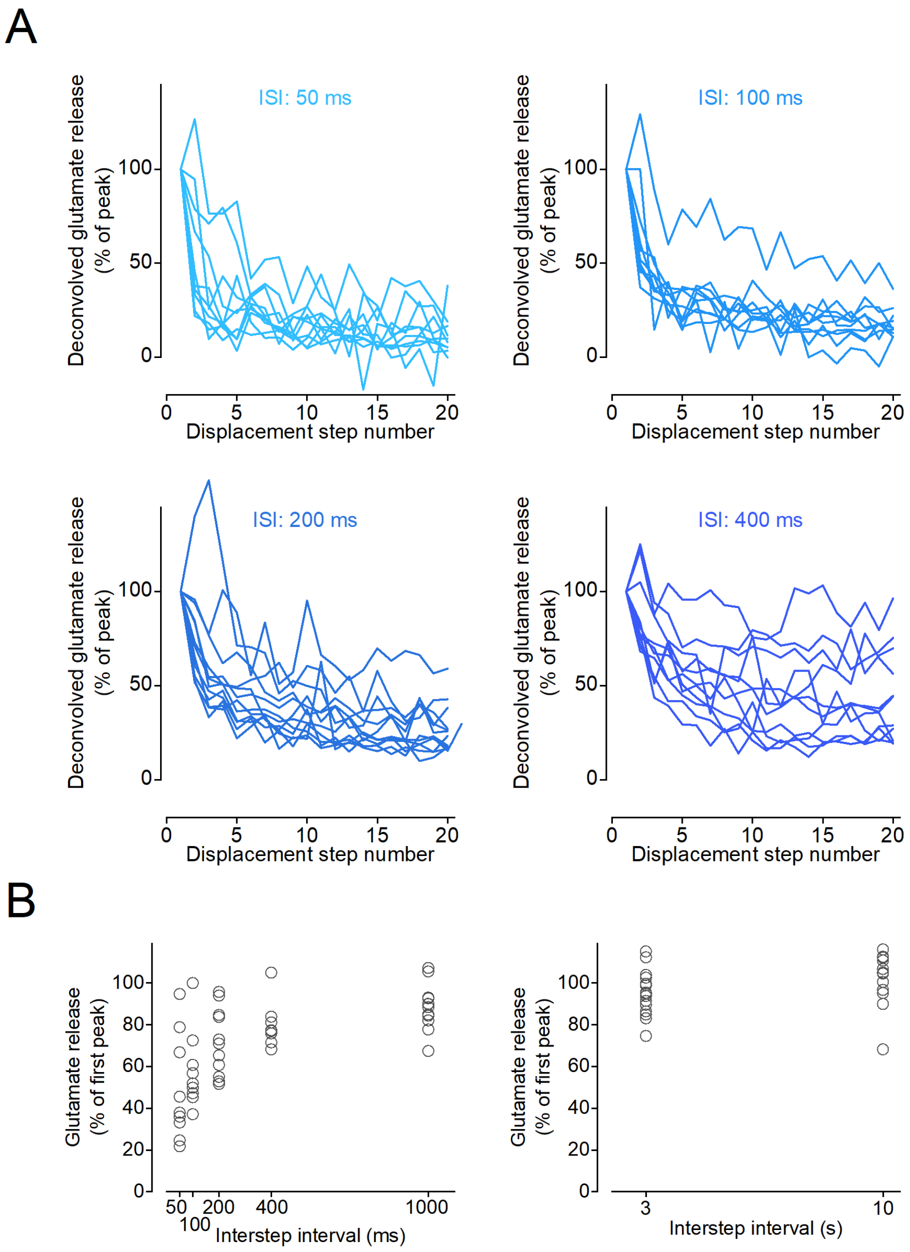

Figure 4. RRP depletion and replenishment in hair cells.

A, Individual recordings used to calculate the average values shown in **Figure 4D**. **B**, Individual datapoints used to calculate the average values in **Figure 4E**.

Figure 5

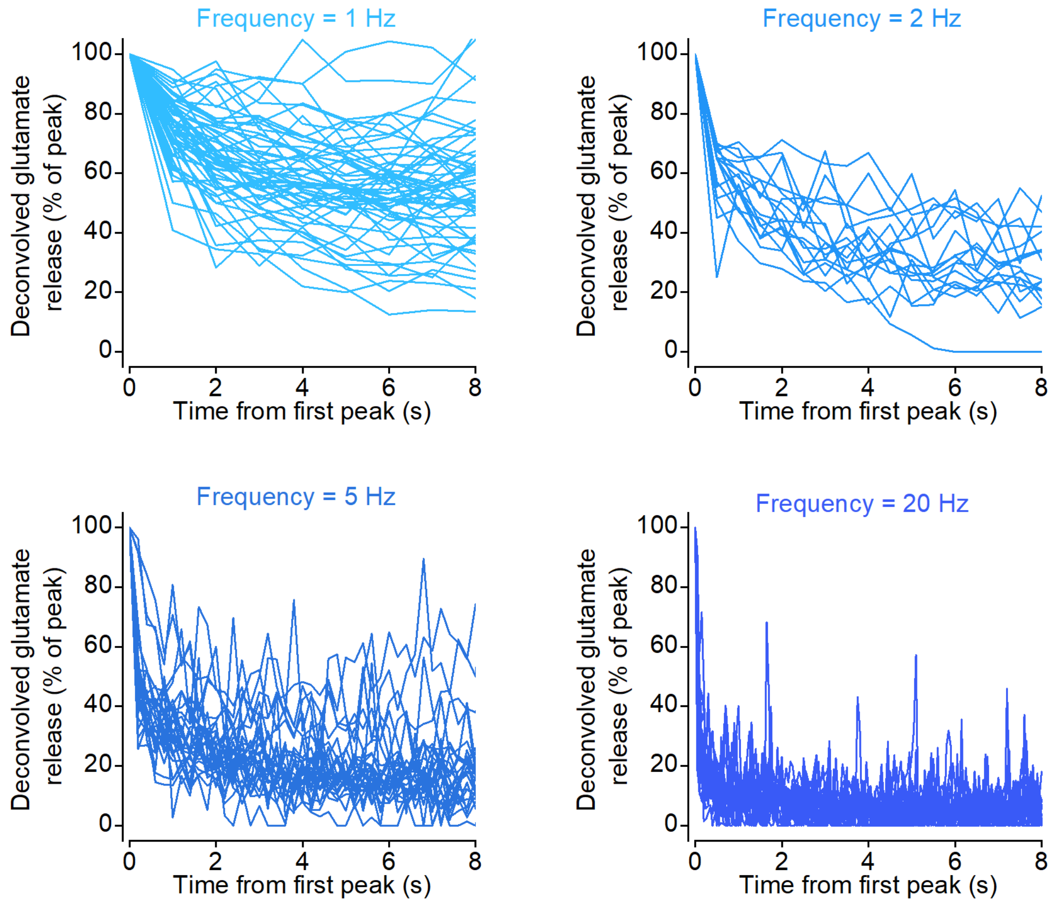

Figure 5. Adaptation of glutamate release in hair cells during periodic stimuli. Individual recordings used to calculate the average values shown in **Figure 5D.**

Figure 6

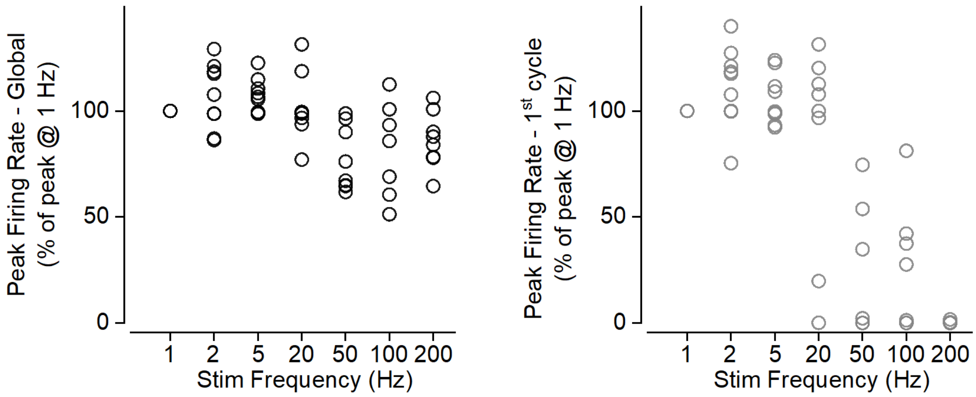

Figure 6. Firing rate adaptation in PLLg neurons during periodic stimuli.

Individual datapoints used to calculate the average Global (left) and 1st cycle (right) Peak firing rate shown in **Figure 6D**.
